# Reassessing Semen Analysis: Clinical Insights Beyond Sperm Count and Motility

**DOI:** 10.1002/rmb2.70096

**Published:** 2026-09-08

**Authors:** Germar‐Michael Pinggera, Pallav Sengupta, Taymour Mostafa, Fahmi Bahar, Aldo E. Calogero, Sindhuja Srinivasan, Ashok Agarwal

**Affiliations:** ^1^ Global Andrology Forum Moreland Hills Ohio USA; ^2^ Department of Urology Medical University Innsbruck Innsbruck Austria; ^3^ Department of Biomedical Sciences, College of Medicine Gulf Medical University Ajman UAE; ^4^ The Department of Andrology, Sexology & STIs, Faculty of Medicine Cairo University Cairo Egypt; ^5^ Faculty of Medicine Muhammadiyah Palembang University Palembang Indonesia; ^6^ Siloam Sriwijaya Hospital Palembang Indonesia; ^7^ Department of Clinical and Experimental Medicine University of Catania Catania Italy; ^8^ Sri Chakra Fertility Chennai Tamil Nadu India; ^9^ Cleveland Clinic Foundation Cleveland Ohio USA

**Keywords:** cryopreservation, male infertility, reproductive technologies, semen, sperm, vasectomy

## Abstract

**Background:**

Semen analysis (SA), recognized by the World Health Organization (WHO) as the cornerstone of male infertility evaluation, remains indispensable in reproductive medicine. However, advances in assisted reproductive technology (ART) and artificial intelligence (AI) have highlighted the limitations of relying solely on conventional semen parameters.

**Objective:**

To critically review the evolving clinical role of SA by integrating conventional assessment with emerging functional, molecular, and computational approaches that improve diagnostic accuracy and individualized patient care.

**Methods:**

A narrative review of contemporary evidence was conducted, focusing on conventional semen parameters, biofunctional sperm testing, omics technologies, AI‐assisted analysis, and broader clinical applications of SA.

**Results:**

Conventional parameters, including sperm concentration, motility, and morphology, remain essential but inadequately reflect fertilizing capacity. Adjunctive assessments, including oxidative stress biomarkers and sperm DNA fragmentation, provide valuable insights into sperm function and reproductive potential. Omics technologies, including genomics, transcriptomics, proteomics, and metabolomics, deepen mechanistic understanding, while AI enhances diagnostic precision, reproducibility, and standardization. Beyond infertility evaluation, SA also supports male contraceptive assessment, natural conception, ART, and patient counseling.

**Conclusions:**

Integrating conventional SA with functional, molecular, and AI‐driven diagnostics provides a comprehensive framework for evaluating male fertility, advancing precision reproductive medicine and personalized clinical management.

## 1 Introduction

Semen analysis (SA) is a cornerstone investigation in the evaluation of male infertility and remains central to clinical decision‐making for infertile couples. Since the publication of the first World Health Organization (WHO) laboratory manual in 1980, SA has been standardized as the reference laboratory test for the evaluation for male infertility [1]. The sixth edition of the WHO manual introduced revised reference limits for conventional semen parameters, reinforcing their role in standardization while simultaneously acknowledging their diagnostic limitations [1]. At the same time, it has become increasingly evident that conventional semen parameters alone are insufficient to capture the biological competence of spermatozoa for natural conception or assisted reproductive technologies (ART).

Beyond its established role in fertility assessment, semen analysis may also provide clinically relevant information regarding broader male health. Semen quality is influenced by advancing age, and age‐related changes in conventional and functional sperm parameters may reflect progressive alterations in reproductive physiology and overall health [2]. Accordingly, this review reassesses the evolving clinical role of SA beyond conventional semen parameters, with an emphasis on its broader applications in reproductive and systemic health. It further examines how biofunctional, omics‐based, and AI‐assisted approaches may complement conventional SA and contribute to more individualized clinical decision‐making.

## 2 Assessing Male Fertility

In clinical practice, SA represents the first‐line investigation for phenotyping male fertility status and stratifying subsequent diagnostic pathways, as male‐related factors contribute to 30% of overall infertility cases and, when combined with female factors, contribute an additional 20% [3]. The identification of sperm alterations in either macroscopic parameters (volume, pH, color, odor, liquefaction, and viscosity) or microscopic parameters [sperm concentration, total sperm count (TSC), total and progressive sperm motility, and percentage of normally shaped spermatozoa], as well as the presence of leukocytes, allows clinicians to direct patients toward appropriate diagnostic and therapeutic pathways [4].

### 2.1 Standardization and Quality Control in Semen Analysis

Different studies report high interlaboratory and inter‐operator variability in SA results, underscoring the need for strict adherence to WHO‐recommended standardized protocols [4]. The 2024 AUA/ASRM guidelines, published by the American Urological Association and American Society for Reproductive Medicine, refer to these new WHO reference values and emphasize compliance with standardized protocols [5].

Therefore, regular quality‐control (QC) procedures, staff training, and adherence to the ISO 23162:2021 standards are recommended to ensure precise results in andrology laboratories [6, 7, 8]. Robust internal quality control (IQC) systems, including analyte‐specific control rules and systematic monitoring of QC data, ensure consistent results and timely corrective actions [7]. Additional key measures include implementation of pre‐analytical and analytical best practices, routine equipment calibration, use of high‐quality reagents, and standardized reporting systems [7, 9]. To further reduce variability and improve diagnostic confidence, laboratories pursue accreditation (e.g., ISO 15189) and increasingly adopt computer‐assisted semen analysis (CASA) systems [10].

### 2.2 Limitations of Conventional Semen Analysis

CASA systems offer significant advantages, such as rapid analysis of semen samples, reduction in subjectivity, better reproducibility, improved sperm kinetics evaluation, and video/image recording capability; however, trained andrology technicians are still needed. Although CASA‐based automated SA systems offer advantages, they present notable limitations, particularly in assessing sperm morphology and in handling samples with extremely high (polyzoospermia) or very low sperm concentrations (severe oligozoospermia) [11]. Viscous samples and spherical‐cell‐containing debris or agglutination remain challenging for CASA. Under ideal circumstances, CASA provides equivalent results for semen parameter analyses, including sperm concentration, motility, and morphology. However, it does not evaluate important aspects, such as capacitation, acrosome reaction, or oocyte fertilization capability, which are essential for achieving pregnancy [12]. Additionally, the expensive nature, lack of FDA/CE approval, and low comparability between various machines restrict their widespread laboratory use [10].

Despite the fundamental role of microscopic examination in male infertility diagnosis, up to 70% of patients with altered sperm parameters lack a precise etiological diagnosis. Even with conventional genetic testing and advances in monogenic sequencing, many cases of oligozoospermia, azoospermia, recurrent pregnancy loss, or ART failure remain unexplained, underscoring the limitations of conventional semen parameters and the need for additional sperm quality assessments [5, 13].

### 2.3 Sperm DNA Fragmentation and Biofunctional Assessment

**FIGURE 1:**
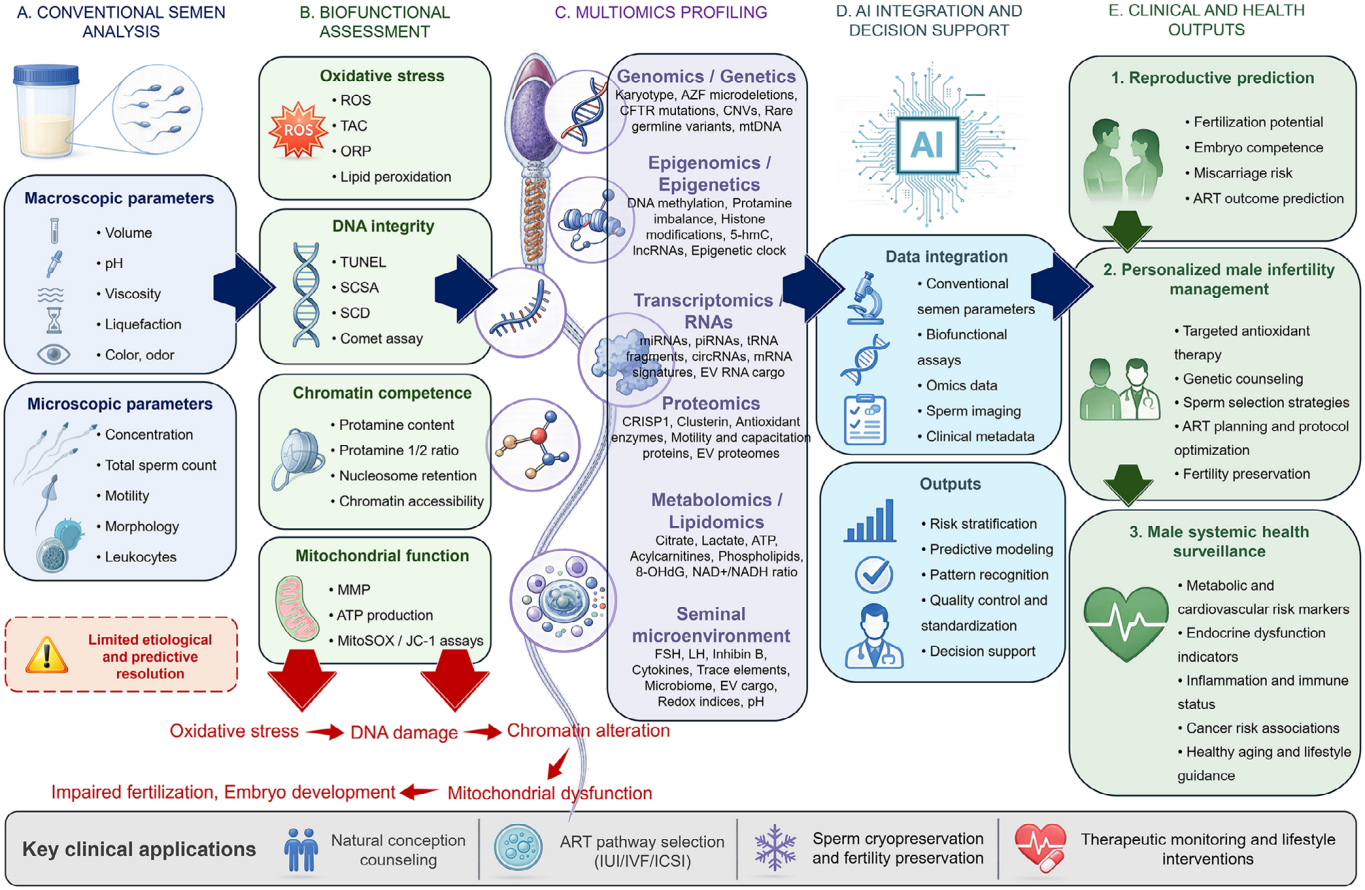
Integrated precision framework for comprehensive male fertility assessment. A proposed precision‐medicine framework that expands conventional semen analysis beyond descriptive sperm parameters toward a multidimensional assessment of male reproductive and systemic health. Conventional semen analysis provides macroscopic and microscopic information but offers limited etiological and predictive resolution. The integration of biofunctional assessments, including oxidative stress, sperm DNA integrity, chromatin competence, and mitochondrial function, enables mechanistic characterization of sperm dysfunction and its downstream consequences. Multi‐omics profiling further enhances diagnostic precision through the evaluation of genomic, epigenomic, transcriptomic, proteomic, metabolomic, and seminal microenvironment biomarkers. AI‐driven platforms can integrate conventional semen parameters, biofunctional assays, sperm imaging, omics datasets, and clinical metadata to support risk stratification, predictive modeling, pattern recognition, quality control, and clinical decision‐making. This integrated framework facilitates reproductive outcome prediction, personalized male infertility management, fertility preservation strategies, assisted reproductive technology (ART) pathway selection, and surveillance of broader male health. AI, artificial intelligence; ART, assisted reproductive technology; AZF, azoospermia factor; CASA, computer‐assisted semen analysis; CFTR, cystic fibrosis transmembrane conductance regulator; CNV, copy number variation; CRISP1, cysteine‐rich secretory protein 1; EV, extracellular vesicle; hCG, human chorionic gonadotropin; HNE, 4‐hydroxynonenal; ICSI, intracytoplasmic sperm injection; IUI, intrauterine insemination; JC‐1, 5,5′,6,6′‐tetrachloro‐1,1′,3,3′‐tetraethylbenzimidazolylcarbocyanine iodide; MMP, mitochondrial membrane potential; mtDNA, mitochondrial DNA; NAD+/NADH, nicotinamide adenine dinucleotide (oxidized/reduced forms); ORP, oxidation–reduction potential; piRNAs, PIWI‐interacting RNAs; ROS, reactive oxygen species; rRNA, ribosomal RNA; SCD, sperm chromatin dispersion; SCSA, sperm chromatin structure assay; TAC, total antioxidant capacity; TEM, transmission electron microscopy; TSC, total sperm count; TUNEL, terminal deoxynucleotidyl transferase dUTP nick‐end labeling; 5‐hmC, 5‐hydroxymethylcytosine; 8‐OHdG, 8‐hydroxy‐2′‐deoxyguanosine.

Because conventional SA cannot detect abnormalities in sperm DNA integrity, chromatin structure, mitochondrial function, oxidative stress (OS), or genetic and epigenetic alterations, additional biofunctional parameters have been proposed to better elucidate the pathophysiology of male infertility (Figure 1). Among these, sperm DNA fragmentation (SDF) has been found to be consistently higher in infertile men than in fertile controls. Elevated SDF has been associated with reduced natural conception rates, impaired embryo development, and increased miscarriage risk, ultimately leading to lower spontaneous pregnancy rates [14, 15, 16]. In ART, including in vitro fertilization (IVF) and intracytoplasmic sperm injection (ICSI), high SDF is also linked to poorer reproductive outcomes, although its impact appears less pronounced than in natural conception [14, 15, 17]. This attenuation may reflect the capacity of the oocyte to repair sperm DNA damage, particularly in younger women with high‐quality oocytes or when testicular sperm retrieval is used [15].

Testicular sperm typically exhibit lower DNA fragmentation than ejaculated sperm, and its use in ICSI for men with high SDF has been associated with improved pregnancy and live birth rates and reduced miscarriage rates [17, 18]. Current international guidelines do not routinely recommend the use of testicular sperm rather than ejaculated sperm for ICSI in non‐azoospermic infertile men, including those with low sperm counts. However, emerging evidence suggests that selected subgroups, particularly men with high sperm DNA fragmentation (SDF) or cryptozoospermia, may benefit from testicular sperm retrieval. In a systematic review and meta‐analysis, Kang et al. evaluated 578 men with cryptozoospermia undergoing ICSI and reported significantly higher good‐quality embryo rates (RR 1.17), implantation rates (RR 1.52), and clinical pregnancy rates (RR 1.74) with testicular sperm compared with ejaculated sperm [19]. Similar results were reported in a recent SRMA by Cano‐Extremera et al. investigating clinical outcomes in terms of fertilization, clinical pregnancy, live birth, and miscarriage rates according to sperm origin [20]. Nevertheless, the level of evidence (LoE) remains low for formulating a general therapeutic algorithm, and single‐center RCTs have not consistently demonstrate higher fertilization, pregnancy, or live birth rates [21]. Given the increased SDF ratio during sperm maturation, strategies to counteract DNA damage, including methylation processes or strand breakage, are needed. Notably, short abstinence (within 1–4 h) before semen collection is associated with reduced SDF. Thus, a second semen collection before ART may serve as a practical intervention [22, 23].

### 2.4 Biomarkers and Multi‐omics Approaches

While clinical and laboratory assessments remain standard, they are subject to intrinsic limitations. Furthermore, SA outcomes are susceptible to a range of physiological influences, such as abstinence duration, systemic diseases, medical conditions, lifestyle habits (e.g., diet and physical activity), and environmental exposures [24, 25, 26]. Given the pronounced variability in semen characteristics, a variety of supplementary biomarkers have been proposed over time. Contemporary research now integrates these biomarkers within multiomics frameworks, enabling a more personalized and mechanistic evaluation of semen quality. Advances in genomics, epigenomics, proteomics, transcriptomics, and metabolomics help clarify the biological pathways behind idiopathic infertility and may improve diagnosis and treatment options (Table 1) [27]. Therefore, accurate evaluation of conventional sperm parameters with biofunctional assessments can improve understanding of male infertility causes, enabling better diagnosis and new therapeutic algorithms.

**TABLE 1:** Integrated overview of biomarkers in male infertility.

Domain	Core Biomarkers (Clinically Relevant)	Advanced/Emerging Biomarkers	Biological/Clinical Insight	Translational Status
Genomic/Genetic	Karyotype, AZF microdeletions, CFTR mutations, FISH (aneuploidy)	mtDNA variants, CNVs, rare germline mutations (DMRT1, TEX11), repeat expansions (AR CAG), regulatory variants	Identifies chromosomal and gene defects affecting spermatogenesis and azoospermia; emerging variants explain idiopathic infertility	Established → Emerging
Epigenetic/Epigenomic	DNA methylation (H19, IGF2), protamine imbalance, SDF (TUNEL, SCSA)	Histone modifications, chromatin accessibility, 5‐hmC, sperm epigenetic clock, lncRNAs, piRNAs	Regulates gene expression, embryo development, and transgenerational inheritance; links OS and infertility	Emerging
Transcriptomic (RNA)	microRNAs (miR‐34c, miR‐19b)	piRNAs, tRNA fragments, circRNAs, mRNA signatures, EV RNA cargo	Reflects sperm maturation, fertilization potential, and embryo viability; enables noninvasive diagnostics	Emerging
Proteomic	Seminal proteins (PSA, transferrin, SEMG1), antioxidant enzymes (SOD, GPX)	CRISP1, LGALS3BP, clusterin, EV proteomes, stress proteins, motility proteins (dynein, tektins)	Indicates sperm function, capacitation, OS response, and ART outcomes; potential predictors of TESE success	Near‐clinical → Emerging
Metabolomic/Lipidomic	Energy metabolites (citrate, lactate), ROS markers (MDA, 8‐OHdG), ATP levels	Acylcarnitines, phospholipids, VOCs, NAD^+^/NADH ratio, metabolic flux analysis	Reflects sperm bioenergetics, mitochondrial function, and OS; explains infertility despite normal SA	Emerging
Microenvironmental/Seminal Fluid	Hormones (FSH, LH, inhibin B), trace elements (zinc), pH, viscosity	Microbiome, cytokines (IL‐6, TNF‐α), EV cargo, redox indices	Influences sperm function, immune balance, and local environment; integrates systemic and reproductive health	Mixed

Abbreviations: 5‐hmC, 5‐Hydroxymethylcytosine; 8‐OHdG, 8‐Hydroxy‐2′‐Deoxyguanosine; AR, Androgen Receptor; ART, Assisted Reproductive Technology; ATP, adenosine triphosphate; AZF, Azoospermia Factor; CFTR, Cystic Fibrosis Transmembrane Conductance Regulator; circRNAs, Circular RNAs; CNVs, Copy Number Variations; CRISP1, Cysteine‐Rich Secretory Protein 1; DMRT1, Doublesex and Mab‐3 Related Transcription Factor 1; EV, Extracellular Vesicle; FISH, Fluorescence In Situ Hybridization; FSH, Follicle‐Stimulating Hormone; GPX, Glutathione Peroxidase; H19, H19 Imprinted Maternally Expressed Transcript; IGF2, Insulin‐Like Growth Factor 2; IL‐6, Interleukin‐6; LGALS3BP, Galectin‐3 Binding Protein; LH, Luteinizing Hormone; lncRNAs, Long NonCoding RNAs; MDA, Malondialdehyde; miR, MicroRNA; mRNA, Messenger RNA; mtDNA, Mitochondrial DNA; NAD^+^, Nicotinamide Adenine Dinucleotide (Oxidized Form); NADH, Nicotinamide Adenine Dinucleotide (Reduced Form); OS, Oxidative Stress; piRNAs, PIWI‐Interacting RNAs; PSA, Prostate‐Specific Antigen; ROS, Reactive Oxygen Species; SA, Semen Analysis; SCSA, Sperm Chromatin Structure Assay; SDF, Sperm DNA Fragmentation; SEMG1, Semenogelin 1; SOD, Superoxide Dismutase; TESE, Testicular Sperm Extraction; TEX11, Testis Expressed 11; TNF‐α, Tumor Necrosis Factor‐Alpha; tRNA, Transfer RNA; TUNEL, Terminal Deoxynucleotidyl Transferase dUTP Nick‐End Labeling; VOCs, Volatile Organic Compounds.

## 3 Monitoring Reproductive Health

Male infertility is increasingly recognized as a public health concern associated with systemic diseases, including malignancies, cardiometabolic disorders, and reduced life expectancy, positioning SA as a potential early indicator of broader health dysfunction [28]. Environmental pollutants and chemical toxicants are major contributors to declining male reproductive and overall health. Heavy metals [29], pesticides [30], endocrine‐disrupting chemicals such as phthalates, bisphenol A (BPA), and dioxins [31, 32], pharmaceuticals [32, 33], and infectious agents exert convergent adverse effects on sperm structure, function, and viability while simultaneously contributing to metabolic, cardiovascular, immune, and endocrine dysfunction.

Epidemiological research has focused on understanding shared risk factors, from some lifestyle exposures (e.g., cigarette smoking, increased body weight, unhealthy diet) to specific underlying pathophysiological mechanisms, such as genetic and epigenetic risk factors. Male infertility should not be viewed solely as an isolated reproductive burden but rather as a potential indicator of impaired overall health status [34]. Healthy diets such as the Mediterranean one are positively associated with increased sperm quality [35].

### 3.1 Male Infertility as a Systemic Health Indicator

Male infertility is rarely considered as a routine biomarker for broader health in clinical practice. Most clinical studies focus on single reproductive variables, neglecting male fertility as a diagnostic variable for longitudinal health monitoring, comorbidity prevention, or early detection of nonreproductive diseases. By contrast, the diagnostic approach to erectile dysfunction (ED) has evolved substantially over the past decade, adopting a more holistic framework that includes screening for metabolic syndrome, cardiovascular disease, mental health disorders, and malignancies. Consequently, ED management now extends beyond symptom control to encompass risk stratification and prevention of associated systemic complications.

Elucidating the long‐term health trajectories of infertile men requires large, longitudinal, and multinational studies. While existing longitudinal data document global declines in male fertility, they provide limited insight into the determinants linking reproductive and systemic health [36]. Targeted longitudinal studies have begun to associate abnormal semen parameters with conditions such as type 2 diabetes mellitus, anemia, hypercholesterolemia, chronic kidney disease, heart failure, benign prostatic hyperplasia, testicular cancer, and adverse lifestyle factors, thereby supporting fertility status as a marker of overall health and a rationale for early interventions, including lifestyle modification, hormonal therapy, and psychosocial support [37, 38]. These observations underscore the need for closer integration between andrology, endocrinology, and internal medicine.

### 3.2 Male Infertility and Chronic Disease Risk

Analogous to ED, which Montorsi et al. characterized as an early vascular marker of coronary artery disease (CAD), preceding its diagnosis in nearly 70% of cases by approximately 40 months [39], male infertility may similarly represent an early window for risk identification and preventive intervention. Supporting this concept, a Danish cohort study of more than 32,000 men demonstrated an increased risk of testicular cancer among those with abnormal semen quality (overall standardized incidence ratio [SIR] = 1.6), with the highest risk observed in azoospermic men (SIR = 3.5), followed by oligozoospermia and asthenozoospermia [40]. Comparable findings were reported in a U.S. cohort, in which azoospermia was associated with a higher overall tumor risk (SIR = 2.9), particularly among younger men, with an eightfold increase in risk observed in those diagnosed before 30 years of age [38].

### 3.3 Klinefelter Syndrome and Long‐Term Health Implications

Male infertility is rarely considered a routine biomarker for broader health in clinical practice. However, the risk of testicular tumors in non‐mosaic Klinefelter syndrome (KS) patients, who commonly present with azoospermia/severe oligozoospermia, hypogonadism, and gynecomastia, is not increased. Nevertheless, KS patients tend to have a higher risk of developing other cancers, such as breast cancer or extragonadal germ cell tumors, especially mediastinal tumors; ultimately, they have a higher prevalence of other disorders, such as vascular diseases, autoimmune disorders, endocrine and metabolic disorders, especially diabetes mellitus, and osteoporosis [41, 42, 43]. The overall prevalence of breast cancer in men is low, and these patients are usually diagnosed in their mid‐60s. However, in 47XXY, the standardized mortality ratio for breast cancer was reported to be approximately 57.8 (95% CI: 18.8 to 135.0), which means that these men had nearly a 60‐fold risk of dying from breast cancer compared with the general male population [44], requiring close long‐term follow‐up [45]. This implies that diagnosis of KS, often based on azoospermia as a presenting symptom, has significant implications for the overall health trajectory of a man. Although KS is associated with a modest reduction in life expectancy by 2.1 [41] to 5 years [46], appropriate long‐term management may mitigate some of the associated health risks (e.g., hormone replacement therapy (if required), regular medical checkups, and lifestyle adjustments).

### 3.4 Obesity, Metabolic Dysfunction, and Semen Quality

Chronic metabolic conditions, including metabolic syndrome and obesity, exert a substantial negative impact on semen quality. Metabolic syndrome is associated with reduced testosterone levels, impaired spermatogenesis, decreased seminal volume, total sperm count, motility, and vitality, and increased SDF levels [47]. Obesity further exacerbates these effects through insulin resistance, functional hypogonadism aggravated by an abnormal testosterone/17β‐estradiol, and pro‐inflammatory cytokines and adipocytokines such as adiponectin, tumor necrosis factor‐α, leptin, interleukin‐1, and IL‐6, which together affect male fertility [48, 49].

### 3.5 Epigenetic Dysregulation and Reproductive Health

Epigenomic research has shown that aberrant DNA methylation and histone alterations in germ cells are correlated with infertility and may elucidate how environmental exposures and OS affect reproductive and overall health [50]. Changes in DNA methylation and noncoding RNAs like miR‐34b, miR‐34c, miR‐122, and miR‐449 have been linked to altered spermiogenesis [51]. Importantly, epigenetic dysregulation has also been implicated in inflammation, metabolic disorders, and oncogenesis, suggesting shared molecular mechanisms underlying infertility and broader disease processes [52, 53].

Finally, men's external appearance plays a role in partner selection. Women's unconscious preferences are shaped by evolutionary mechanisms favoring genetic fitness and offspring health. They are attracted by their physical, pronounced masculine appearance and sexual attractiveness, which signal health, strength, and reproductive viability and becomes important especially during womens most fertile phase of their cycle [54]. This subconscious bias toward physical markers of attractiveness and appearance aligns with overall indicators of health, reflecting a preference for partners perceived to carry “good genes” and to provide long‐term support [55]. These integrated biological and genetic factors guide mate selection strategies to maximize reproductive success and offspring health [56, 57].

## 4 Semen Analysis and Aging

Aging is a natural and inevitable process marked by physiological changes in the body, including the reproductive system [58]. Age affects fertility in women, and those over 35 years of age experience a decrease in the production of good‐quality oocytes [59]. Although the decline in male reproductive potential is less abrupt, a similar phenomenon, referred to as advanced paternal age (APA), has increasingly been recognized as an important factor influencing male fertility and offspring health. Male fertility is generally regarded as relatively stable until the mid‐30s, after which gradual declines in sperm concentration, motility, and morphology are often observed, particularly after 40 years of age [37]. These changes are associated with progressive impairment of testicular function, affecting both steroidogenesis and spermatogenesis [60]. Furthermore, APA is characterized by increased gonadotropin levels and a higher risk of benign prostatic hyperplasia; a decrease in ejaculate volume, testicular volume, and Leydig and Sertoli cell numbers; changes in testicular vascularization resulting in testicular fibrosis; and increased OS due to higher reactive oxygen species (ROS) production leading to increased lipid peroxidation [61].

In addition to influencing sperm parameters, ROS negatively affects the fertilization capacity of spermatozoa. Men older than 50 years have a 4.58 times greater risk of having an increased SDF rate than younger men between the ages of 21 and 30 [61]. Furthermore, this increase raises the risk of genetic mutations, which not only negatively impact male fertility but also increase the risk of miscarriages, congenital abnormalities, metabolic, neurologic, and psychiatric disorders in offspring [62, 63]. Indeed, several large cohort studies have demonstrated that children of older fathers have a higher risk of neurodevelopmental disorders, including autism spectrum disorder [64], schizophrenia, and attention‐deficit/hyperactivity disorder, as well as certain metabolic conditions. The underlying mechanisms associated with aging are thought to involve *de novo* mutations, changed sperm DNA‐methylation grade, and epigenetic drift.

### 4.1 Advanced Paternal Age (APA) Effects

New social conditions and progress in ART have contributed to the delay in parenthood in developed countries. While the effects of maternal age have been widely studied, showing that women over 35 years have a higher risk of infertility, pregnancy complications, spontaneous abortion, congenital anomalies, and perinatal complications, APA has historically garnered comparatively less attention. APA at first pregnancy has risen significantly over the last decade owing to various variables, including a longer life expectancy, more access to contraception, and later marriage [2].

#### 4.1.1 Effect of APA on Semen Parameters

In their systematic review, Nguyen‐Powanda and Robaire pointed out that most studies showed a general reduction in the proportion of sperm with normal morphology with APA [65]. Lately, Demirkol et al. retrospectively analyzed SA records of 500 infertile men categorized as 20–29, 30–34, 35–39, 40–44, and 45–55 years [66]. While no linear relationship was observed in semen volume, concentration, and total sperm count with age, a linear decline was observed in progressive motility, vitality, and morphology parameters with advancing age.

#### 4.1.2 Effect of APA on Sperm DNA Fragmentation

Aged men display higher levels of DNA damage, in part due to OS and an increased number of mitotic divisions during spermatogenesis [67]. A study of 66 males (aged 20–57 years) showed that sperm from men > 35 years had significantly more DNA breaks than younger men. Furthermore, aging results in impaired sperm chromatin integrity and increased breaks in sperm DNA in infertile men compared to younger men [68]. Lately, Kadoch et al. retrospectively explored 4250 consecutive semen samples from infertile patients, stratified into seven age groups: < 26, 26–30, 31–35, 36–40, 41–45, 46–50, > 50 years [69]. A positive correlation was observed between paternal age and SDF that remained relatively constant until the age of 35 but increased significantly beyond age of 35.

#### 4.1.3 Effects of APA on Fertility and Progeny Outcome

Previously, Hassan and Killick demonstrated a fivefold increase in time to pregnancy in couples where the male partner was > 45 years old and that men were 4.6 times less likely to impregnate their female partners after 1 year of regular unprotected sex compared to younger men (< 25 years old) [70]. In addition, men of APA were 12.5 times more likely to be among the men who took over 2 years to attain pregnancy with their partners. OS paired with aging can present other reproductive risks due to the extensive amount of damage seen in aged sperm being linked with a higher risk of miscarriages, preterm births, late fetal death, and low birth weight [71].

#### 4.1.4 Effects of APA on Sperm Methylation Landscape

Jenkins et al. reviewed studies reporting age‐associated sperm epigenetic changes, including DNA methylation, histone modifications, and noncoding RNAs [72]. In addition to chromatin decondensation, age‐associated alterations in sperm DNA methylation have been associated with reduced fertility and impaired embryonic development. Similarly, Ashapkin et al. pointed out that age affects all epigenetic mechanisms, including DNA methylation, histone modifications, and profiles of small noncoding RNAs [73]. Successful development of the human sperm epigenetic clock indicates that at least some CpG sites exhibit a linear relationship between methylation levels and age. Although most epigenetic modifications are erased in the early mammalian embryo, there is growing evidence that an altered offspring epigenome and phenotype are linked with APA due to the father's sperm accumulating epigenetic changes with time. These findings raise concerns about delayed fatherhood and age‐associated changes in the sperm epigenome that may compromise the reproductive health of fathers and transfer altered epigenetic information to subsequent generations. In their retrospective study, Lahimer et al. concluded that men aged> 40 years are at higher risk of elevated SDF and lower methylation levels [74]. Mohammed and Maged suggested that APA increased SDF, chromatin decondensation, and global DNA methylation levels in human spermatozoa, which negatively affects the ICSI outcomes [75].

#### 4.1.5 Effects of APA on ICSI Outcome

Few studies have reported no significant effect of APA on ICSI outcome. In their study, Ghuman et al. showed that live birth and miscarriage occurrence following ART were not adversely affected by increasing sperm donor age of up to 45 years [76]. Similarly, Tsai et al. addressed that there is insufficient evidence to demonstrate an unfavorable effect of APA on the fertility outcomes for TESE‐ICSI [77]. Additionally, Elbardisi et al. reported that APA affects sperm morphology, fertilization, and embryo cleavage in ICSI but does not appear to affect clinical pregnancy, miscarriage, or live‐birth rates [78]. In line, Kong et al. found no effects of male age on pregnancy or neonatal outcomes in infertile couples following their first frozen–thawed embryo transfer cycles when females were younger than 36 years old [79].

However, many studies have addressed the negative impact of APA on ICSI outcome. Previously, Bartolacci et al. retrospectively analyzed the effect of APA on reproductive outcomes in 1266 ICSI cycles [80]. The male factor neither affected the top‐quality blastocyst formation rate nor the ongoing pregnancy rate. McPherson et al. evidenced the negative associations between paternal age and both viable pregnancies and live births, such that the probability of pregnancy was 10% further reduced for women who were 35 years with a partner over 40 years vs. women aged 35 years with a partner under 30 years [81].

In their study, Datta et al. pointed out that in fresh IVF cycles there was no impact of male age on LBR in any female‐partner age group when ICSI was performed in either the presence or the absence of male infertility [82]. These were the largest clinical data to support the laboratory evidence of the ability of oocytes from young women to reverse the age‐related deterioration of sperm quality. Similarly, Atik et al. highlighted reduced odds of live birth, clinical pregnancy, and increased odds of miscarriage in those of paternal age 51 years compared with fathers ≤ 35 years [83]. Maia et al. pointed out that a discernible trend toward a negative correlation with paternal age was observed, signifying that higher paternal age was linked to lower fertilization rates (*p* = 0.004) [84].

Therefore, future research directions are identified to further comprehend the long‐term effects of advanced paternal age on offspring health within the context of ART, aiming for the best possible outcomes for couples and their children.

## 5 Sperm Banking

Sperm cryopreservation is one of the simplest methods for preserving male fertility and is a fundamental element of ART [85]. It is useful for patients with malignant diseases requiring radiotherapy or chemotherapy, as well as those with testicular tumors scheduled for orchiectomy. Additional indications are fertility preservation prior to vasectomy, gender‐affirming surgery, or testosterone replacement therapy.

### 5.1 Cryopreservation in Men With Elevated SDF and OS

An additional clinical question concerns whether cryopreservation should be considered in men with high SDF or elevated OS. These conditions are associated with reduced fertility, poorer ART outcomes, and recurrent pregnancy loss. In some patients, sperm DNA damage may worsen over time or following medical treatments. Cryopreservation may therefore be considered as a precaution in selected cases.

High SDF and OS are linked to several causes of male infertility. These include inflammation, hormonal imbalance, and lifestyle factors such as cigarette smoking. Clinical guidelines generally recommend treating the underlying causes first. Lifestyle changes and medical therapy may improve OS and reduce SDF before fertility treatment. Cryopreservation itself can also affect sperm DNA integrity. Several studies have shown that SDF increases after thawing compared with fresh samples. Cryoinjury occurs during freezing and thawing. Rapid cooling to minus 196°C and ice crystal formation can damage sperm membranes and cellular structures. As a result, up to half of spermatozoa may lose structural integrity after thawing [86]. Therefore, prophylactic sperm cryopreservation in men with high SDF may be justified for fertility preservation but is less important when the underlying causes are modifiable, such as lifestyle‐related factors. Proper counseling that cryopreservation is a fertility preservation strategy, not a treatment for high SDF, and that post‐thaw sperm may have increased DNA damage is mandatory.

### 5.2 Social Sperm Freezing

Another question concerns elective or social sperm freezing. Social oocyte cryopreservation allows women to preserve fertility before the natural age‐related decline in oocyte quality [87]. The Royal College of Obstetricians and Gynaecologists (RCOG) recommends that elective oocyte freezing is most effective when done at a younger age, preferably before 35 years [87]. Freezing oocytes before the age of 35 years yields significantly higher live birth rates. For example, when at least 20 mature oocytes are frozen before age 38, there is up to a 60%–70% chance that a woman could have a baby later [88, 89]. But in healthy men, daily sperm production—typically ranging from 150 million to 275 million spermatozoa—and the concept of social sperm freezing is different from that of oocyte freezing in women. Correctly, the expected decline in parameters such as sperm motility, progressive motility, morphology, and higher SDF worsen significantly with age, especially after 35–40 years [70]. Furthermore, data show that the reduction in daily sperm production might be 30% or greater in men older than 50 years [58]. Notably, these men have an even 4.58‐fold greater risk of having an elevated SDF rate than younger men aged 21–30 years [61]. However, cryopreservation and post‐thaw damage are unlikely to fully account for such declines, making them insufficient justifications in men.

### 5.3 Cryopreservation in Severe Male Factor Infertility

The evaluation differs in ART settings, where sperm cryopreservation is advantageous when the male partner is unavailable on the day of oocyte retrieval or insemination, as previously frozen sperm can be thawed and used during ART procedures. Furthermore, the available spermatozoa can be cryopreserved in cases of severe oligozoospermia or cryptozoospermia. Under the most severe conditions, such as obstructive azoospermia (OA) and nonobstructive azoospermia (NOA), cryopreserved spermatozoa might be useful to optimize female ovarian stimulation [90]. In contrast, when surgical sperm retrieval has failed for NOA, the option of using cryopreserved sperm donated by commercial sperm banks becomes available. This option, however, may not be available to everyone because of legal issues in some countries and the opposition of some religious groups.

## 6 Guide to ART

Although the predictive value of conventional SA is limited, the female partner's age and semen analysis findings remain central to treatment selection in infertile couples. Many factors influence the type of ART treatment, whether it is intrauterine insemination (IUI), IVF, or ICSI, such as the couple's age, infertility duration, ovarian reserve, and ovulation potential. The decision includes consideration of the quality of the semen, although there are no universally defined cutoff values. An IUI is more likely to be successful when the total motile sperm count (TMSC) is more than 10 million, whereas a TMSC of 1–5 million is more appropriate for IVF, and less than 1 million is appropriate for ICSI. Beyond concentration, motility and morphology are also decisive in defining the ART to be applied. Importantly, these cutoff values are dictated by regional and population characteristics, and IUI decision making is vastly different from IVF or ICSI [91]. The clinical relevance of SA in ART was demonstrated by Villani et al. in a large single‐center study. They reported that sperm motility is the best predictor of fertilization rate in IVF. Morphology shows the closest relationship with fertilization and pregnancy outcomes in ICSI [92].

A meta‐analysis showed that isolated teratozoospermia does not significantly reduce clinical pregnancy rates in ART cycles regardless of whether ICSI is used. Although sperm morphology is a vital predictor of pregnancy after IVF, previous studies have not unequivocally displayed whether teratozoospermia worsens the likelihood of pregnancy after IVF. For patients with previous IVF failure and those with compromised sperm parameters, ICSI may allow fertilization to be achieved. Previously, sperm morphology was thought to have a greater impact on clinical pregnancy rates after ICSI than other conventional sperm parameters. However, whether ICSI enhances ART outcomes in patients with teratozoospermia is unclear since a single morphologically normal spermatozoon can be selected from a cohort of abnormal spermatozoa [93].

## 7 Research and Epidemiological Studies

Over recent decades, a global decline in fertility has paralleled a reduction in total sperm count, raising concerns about male reproductive health at a population level [94]. This trend is multifactorial and reflects the combined effects of industrialization, environmental pollution, and shifts in lifestyle, including smoking, alcohol consumption, poor diet, obesity, and exposure to heat and radiation [95]. These factors disrupt spermatogenesis through several interconnected mechanisms, including impairment of the hypothalamic–pituitary–testicular axis, epigenetic alterations such as abnormal DNA methylation, disturbances in seminal micronutrient balance, and direct structural damage to spermatozoa [96, 97].

Despite its central role in clinical practice, SA has important limitations that restrict its ability to fully characterize male fertility. Results remain subject to considerable inter‐ and intra‐observer variability, and conventional parameters provide little insight into underlying molecular or functional defects [7]. Moreover, reference thresholds often overlap between fertile and infertile men, and normal semen parameters do not necessarily reflect normal fertilizing capacity [1, 98]. As a result, the etiology of many cases of male infertility remains unexplained [99]. These limitations have driven the development of more advanced diagnostic approaches aimed at assessing sperm function at the biochemical and molecular level.

### 7.1 Measurement of ROS, Total Antioxidant Capacity (TAC), Oxidation–Reduction Potential (ORP), and ROS‐TAC Score

Increasing attention has been directed toward OS, a key mediator of sperm dysfunction. Laboratory assessment of OS includes measurement of ROS, total antioxidant capacity, oxidation–reduction potential, and combined indices such as the ROS‐TAC score [12, 100]. These markers can help identify patients who may benefit from targeted antioxidant strategies.

A range of laboratory techniques has been proposed to quantify OS, each with distinct advantages and limitations. Direct methods, such as chemiluminescence and electron spin resonance, offer high sensitivity for detecting reactive species but are often technically demanding, time‐consuming, and limited by sample requirements or rapid molecular interactions [101]. Other approaches, including nitroblue tetrazolium and cytochrome C reduction assays, provide indirect estimates of oxidative activity but either lack standardized interpretation or fail to detect intracellular processes under certain conditions [102]. Fluorescence‐based methods can evaluate sperm structures such as the acrosome, although signal instability and interpretation challenges limit their routine use [103, 104].

Indirect assessments complement these techniques by evaluating downstream effects of OS, including lipid peroxidation, antioxidant capacity, and DNA damage. Among these, oxidation–reduction potential has emerged as a practical indicator of the overall balance between oxidants and antioxidants, offering a more integrated measure of oxidative status. Thus, these advances reflect a shift from purely descriptive semen analysis toward a more mechanistic understanding of male infertility. However, despite their promise, many of these tests remain limited by variability, lack of standardization, and uncertain clinical thresholds, highlighting the need for further validation before widespread clinical adoption.

### 7.2 Assessment of SDF

SDF reflects structural damage within sperm chromatin and includes single‐ and double‐strand DNA breaks [105]. When DNA damage exceeds the repair capacity of the oocyte, it may impair embryo development and is associated with reduced natural and ART pregnancy rates, abnormal embryogenesis, and recurrent pregnancy loss [106]. The underlying mechanisms are complex and may involve abortive apoptosis, defective chromatin maturation, and OS [107].

To better characterize sperm DNA integrity, several laboratory methods have been developed, each targeting different aspects of chromatin damage. The TUNEL assay directly labels DNA strand breaks and reports the proportion of spermatozoa with fragmented DNA. It can be applied to both fresh and frozen samples and is particularly useful in cases of severe oligozoospermia. However, variability in laboratory protocols and the lack of universally accepted threshold values limits its comparability across centers [108, 109, 110, 111].

The sperm chromatin structure assay (SCSA) evaluates chromatin susceptibility to denaturation and provides an indirect measure of DNA damage through the percentage of spermatozoa with high DNA stainability. This reflects incomplete chromatin compaction due to abnormal protein retention. SCSA benefits from standardized protocols and established clinical thresholds, but its use is restricted by the need for specialized equipment and larger sample volumes [110]. Another widely used method is the sperm chromatin dispersion test, which assesses DNA integrity based on the formation of nuclear halos after denaturation. Spermatozoa with intact DNA display large halos, whereas fragmented DNA results in reduced or absent dispersion. Although this test is relatively simple, its interpretation is time‐consuming and may vary between observers [112].

The comet assay provides a more detailed evaluation by measuring DNA migration during electrophoresis. Fragmented DNA forms a characteristic tail, while intact DNA remains in the head region. This technique allows single‐cell analysis and can be performed with small sample volumes. However, manual interpretation can be labor‐intensive and subject to variability, although computerized image analysis may improve standardization [110, 113].

Although these assays have expanded the diagnostic evaluation of male infertility, their clinical integration remains limited by differences in methodology, interpretation, and threshold values. Further standardization and validation are required before routine implementation in clinical practice.

## 8 Prevasectomy and Postvasectomy Analysis

Of all the available methods, vasectomy is a permanent, simple, and safe method of contraception and is currently used by approximately 5% of men worldwide [114]. It involves bilateral ligation or cutting of the vas deferens to prevent the release of spermatozoa from the testes. Despite its permanence, about 6%–10% of vasectomized men later seek fertility restoration [115]. This can be achieved through microscopic vasectomy reversal (VR) [116]. SA remains the fundamental diagnostic test to verify the success of vasectomy or assess fertility recovery after VR.

Several guidelines provide their own criteria for vasectomy success based on postvasectomy SA assessment [117, 118, 119]. VR success is generally defined clinically by the reappearance of spermatozoa in post‐reversal semen samples, typically observed several weeks to months after surgery. However, sperm parameters may vary widely, and SA helps guide patients prognosis and further management [119]. Majzoub et al. discovered that semen parameters sufficient for pregnancy after VR are below the WHO normal reference values, so VR patients will likely succeed at conceiving even though they have lower sperm concentration, motility, and morphology compared with the larger fertile population [120].

Later proteomic assessments identified promising protein biomarkers in vasal fluid predictive of VR success and sperm presence such as CRISP‐1 and lipocalin‐type prostaglandin D synthase (L‐PGDS), which could improve diagnostics if confirmed [121]. Post‐reversal Seminal antisperm antibody (ASA) levels may not significantly correlate with pregnancy rates or semen parameters, suggesting that ASAs alone are insufficient to predict fertility outcomes after VR [122]. The role of ROS measurements seems conceptually attractive but has been insufficiently investigated to evaluate postoperative outcomes.

## 9 Counseling and Decision‐Making

The diagnosis of infertility does not depend exclusively on SA results [123]. Poor SA results do not rule out the likelihood of pregnancy. In contrast, normozoospermic samples do not guarantee pregnancy. Therefore, TMSC assessment is used more frequently due to its stronger correlation with the probability of spontaneous pregnancy [124].

Patients with mild‐to‐severe abnormalities may still benefit from ART interventions. However, in conditions where natural conception is impossible (e.g., De la Chapelle syndrome), options are limited to donor spermatozoa or adoption. Unfortunately, donor insemination is not feasible in Muslim countries [125, 126].

Learning that conception may not be possible can be emotionally distressing for individuals and couples facing infertility. Bad news relates not only about life‐threatening situations, but also to circumstances that extinguish hope and cause worries about the future [127]. Counseling to ease the pain of infertility is certainly not a simple matter. Several structured communication protocols for this purpose, such as ABCDE [128], BREAKS [129], and SPIKES [130].

## 10 Prospects and Emerging Technologies

The role of SA in male genital tract dysfunction and infertility, and its role in ART will continue to evolve in the coming years. The testing method may vary with the introduction of home analysis kits. In addition to SA core measures (i.e., semen volume, sperm concentration, motility, and morphology), other semen analytic methodologies are constantly being developed to provide additional information on male reproductive function. Some of these methods include CASA, SDF, sperm morphometry, sperm aneuploidy rate, sperm epigenetic profile, and sperm RNA analysis [131].

Still, the discussion on the prospects of SA is incomplete without mentioning the role of AI. Other approaches include the intracellular pH assessment to evaluate the ability to predict fertilization success in normozoospermic men, sperm videos to evaluate sperm motility, and sperm selection for ICSI or IVF. These computer‐based technologies will revolutionize the approach to SA and andrology laboratory procedures (Figure 1). They could also improve our knowledge in the diagnosis and treatment of male infertility, thus improving patient clinical benefit [132].

### 10.1 Role of Artificial Intelligence (AI)

Artificial intelligence is increasingly used in medicine to improve healthcare outcomes using algorithms created through data analysis, paving the way for this technology to increasingly become a crucial part of contemporary healthcare [133]. AI applications are currently being developed to assist physicians in surgical, outpatient, and inpatient settings and to support the delivery of patient‐centered precision medicine.

The growing amount of data available makes the use of AI in its various applications increasingly necessary. These include machine learning, artificial neural networks, and deep learning, which show immense potential in reproductive medicine. Many aspects of reproduction can benefit from these valuable applications resulting from big data analysis. These aspects involve sperm classification, oocyte and embryo selection, outcome prediction, robotic surgery, clinical decision‐making systems, cost‐effectiveness, sperm selection, and assessment of sperm motility and morphology [134, 135].

Most manual calculations of sperm parameters are time‐intensive, highly subjective, require a prolonged training process, and are sensitive to human error and intra‐operator variability [10]. Before the introduction of CASA systems, conventional SA was sometimes inaccurate and inconsistent [136]. These automated systems now use sophisticated cameras and software to analyze microscopic results and, more precisely, determine sperm parameters. They may also help evaluate kinematic sperm parameters and hyperactivation [10, 137, 138]. CASA systems are more objective and reproducible than assessments using manual methods [139].

Finelli and colleagues identified a strong correlation between motility and sperm concentration when comparing manual and CASA‐based SA. This relationship was observed only for samples with medium sperm concentrations. Otherwise, sperm morphology remains problematic due to the wide variety of abnormalities and classifications [10]. These findings are consistent with those of a prior study comparing AI‐based optical microscopy in the LensHooke X1 PRO with IVOS CASA and CSA systems for evaluating normal sperm morphology, which identified significant discrepancies among the three methods [140].

By using a dataset comprising 21,600 images with 12,683 annotated unstained sperm cells and employing a ResNet50 transfer learning model, a deep neural network optimized for image classification tasks, Jaruenpunyasak et al. achieved a precision of 0.95 and a recall of 0.91 in distinguishing normal from abnormal sperm morphology [141]. Importantly, their AI algorithm was tested and validated on unstained, live sperm, thus avoiding the staining and sample preparation steps, which are typically needed in conventional methods and are time‐consuming steps. The advancement of AI‐assisted CASA remains highly dependent on the availability and quality of data input. However, to date, only a limited number of publicly accessible sperm image datasets have been available, including the HuSHeM dataset [142], the Human Sperm Morphology Analysis Dataset (HSMA‐DS; 1457 images) [143] and the Visem dataset, a multimodal collection comprising 85 videos along with associated metadata [144]. The release of larger, freely available datasets, such as the SIVA database [145] is anticipated to further increase the sensitivity and accuracy of AI‐integrated CASA systems, particularly in capturing morphological features unique to sperm cells. Lately, an automated AI model has been developed using optical microscopic technology for the assessment of sperm concentration, motility, and seminal pH [140, 146]. However, according to Engel and colleagues, the quality of SA performed manually and in AI analysis using comparable devices is similar [147].

Along with their study, Bachelot and colleagues suggested that in addition to the essential semen quality parameters for the assessment of male fertility, other vital factors should be considered. On the contrary, emerging AI techniques may offer a unique opportunity to tailor all these parameters and provide personalized care [148].

### 10.2 Omics Technologies in Male Infertility

Omics aims to analyze structural and molecular functions across multiple biological levels, including genes (genomics), transcripts (transcriptomics), proteins (proteomics), and metabolomes (metabolomics) [149]. Such approaches enhance our ability to elucidate the etiology of male infertility.

#### 10.2.1 Genomics

Approximately 4000 genes associated with the meiotic and postmeiotic phases of spermatogenesis are already expressed during the premeiotic phase, suggesting that precursor cells already contain the genes necessary for cellular differentiation [150]. The genetic basis of male infertility can be a consequence of gene mutations, chromosomal abnormalities, copy number variations, Y chromosome microdeletions, or monogenic and polygenic disorders [151].

Some genes that play crucial roles in spermatogenesis map to the AZF region on the Y chromosome (Yq11.2). Therefore, their microdeletion can lead to genetic abnormalities that cause male infertility. AZF genes encode 27 proteins [152] that play essential roles in spermatogenesis. AZF has three known regions (AZFa, AZFb, and AZFc). Males who have either a full AZFa, AZFb, or combined AZFab deletion are often azoospermic. However, azoospermic patients who have an AZFc deletion have a 67% probability of having testicular spermatozoa [153]. Additionally, several other genes are important for spermatogenesis, and their mutations can cause obstructive azoospermia, such as in the case of *CFTR* gene mutations, or spermatogenic failure, such as mutations in the following genes: *DMC1, DNAH6, MAGEE2, SLC26A8, TEX11*, and *ZMYND15*, among others [154].

#### 10.2.2 Transcriptomics

Gene readouts during the transcriptional phase are called transcripts. The transcriptome is the collection of all the gene readouts present in the cell [155]. Studies by Zhao and colleagues [156] on transcriptomics have shown that spermatogenesis is a process necessitating controlled regulation of different transcriptional aspects, and long noncoding RNAs (lncRNAs) are critical for normal spermatogenesis and sperm function. Numerous articles have reported the importance of transcriptomic analysis in identifying genes crucial for spermatogenesis.

#### 10.2.3 Proteomics

Proteomics (the study of protein expression [157]) applied for seminal profiling under different conditions can provide important insights into the molecular mechanisms underlying sperm abnormalities. Maintenance of redox balance is essential for preserving sperm function, including after cryopreservation. Notably, seminal proteomic profiling identified differential expression of 99 proteins, highlighting potential biomarkers associated with sperm cryotolerance [151]. Analysis of seminal plasma of infertile patients and fertile men who underwent varicocelectomy showed that 11 proteins, such as serine protease inhibitor A5 (*SERPIN A5*), in the seminal plasma of fertile men were upregulated after varicocelectomy [158].

#### 10.2.4 Metabolomics

Metabolomics is an essential reflection of gene expression and therefore represents a certain phenotype. In this regard, the main metabolic alterations in the blood plasma of infertile patients with various sperm parameter abnormalities included increased levels of energy‐related metabolism (tricarboxylic acid cycle, pyruvate metabolism, glyoxylate, and dicarboxylate metabolism, glycine, serine, threonine metabolism, and saturated fatty acid metabolism) and increased levels of glutathione metabolism, which is related to OS [159].

Metabolites that have been shown to impact spermatogenesis and, therefore, SA outcome include isopentenyl pyrophosphate, acylcarnitine, 2‐phosphoglyceric acid, and γ‐glutamyl‐Se‐methylselenocysteine. The alteration of various metabolic pathways has also been reported to play a role in infertility. The main ones are the citric acid cycle, alanine, aspartate, and glutamate metabolism. Metabolic profiling is becoming essential for modern reproductive medicine and, hence, could be used to detect altered metabolic pathways [159].

## 11 Conclusions

SA remains a fundamental tool in the evaluation of male infertility, but its role must be redefined beyond conventional parameters. Traditional measures such as sperm count, motility, and morphology, while essential, are insufficient to fully capture the biological competence of sperm or explain a large proportion of unexplained male infertility. The integration of biofunctional markers, particularly SDF and OS, provides deeper insight into sperm quality and reproductive potential. Increasing evidence also supports the concept that semen quality reflects broader aspects of male health, linking reproductive function with metabolic, cardiovascular, and oncological risks. In this context, SA should be considered not only a diagnostic test for infertility but also a potential indicator of systemic health. Emerging technologies, especially AI and omics‐based approaches, are expected to transform SA from a descriptive assessment into a more predictive and personalized diagnostic platform (Figure 1). These advances may improve standardization, reduce variability, and help uncover underlying molecular mechanisms relevant to targeted management strategies. Moving forward, the clinical utility of SA will depend on its integration with functional, molecular, and computational tools. Such an approach will enable more precise and individualized management of male infertility and may ultimately position SA as a key biomarker in both reproductive and overall male health.

## Funding

The authors have nothing to report.

## Conflicts of Interest

The authors declare no conflicts of interest.

## Data Availability

Data sharing not applicable to this article as no datasets were generated or analyzed during the current study.
